# Bulimia Nervosa – medical complications

**DOI:** 10.1186/s40337-015-0044-4

**Published:** 2015-04-03

**Authors:** Philip S Mehler, Melanie Rylander

**Affiliations:** Department of Medicine, University of Colorado Health Sciences Center, ACUTE at Denver Health, Denver, CO, 777 Bannock Street, MC4000, Denver, CO 80204 USA; Department of Medicine, University of Colorado Health Sciences Center, Eating Recovery Center, 7351 E Lowry Blvd, Suite 200, Denver, CO 80230 USA

**Keywords:** Bulimia nervosa, Medical, Complications, Self-induced vomiting

## Abstract

As with anorexia nervosa, there are many medical complications associated with bulimia nervosa. In bulimia nervosa, these complications are a direct result of both the mode and the frequency of purging behaviours. For the purposes of this article, we will review in detail the many complications of the two major modes of purging, namely, self-induced vomiting and laxative abuse; these two account for more than 90% of purging behaviours in bulimia nervosa. Some of these complications are potentially extremely dangerous and need to be well understood to effectively treat patients with bulimia nervosa. Other methods of purging, such as diuretic abuse, are much less frequently utilized and will only be mentioned briefly. In a subsequent article, the treatments of these medical complications will be presented.

## Background

Self-induced vomiting is the most frequently used purging method which patients with eating disorders revert to in order to compensate for binging behaviour and in order to lose weight. The medical complications of self-induced vomiting come to clinical attention in a variety of ways and manifest themselves through physical exam findings as well as unique laboratory anomalies. This section of the article will review the medical complications of self-induced vomiting. Some of the following information is based on expert clinical opinion, with some based on cohort studies.

## Review

### Cutaneous manifestations

The cutaneous effects of self-induced vomiting are either a reflection of starvation or the act of inducing emesis. Where empirical evidence (e.g., from randomised controlled trials) is lacking, information is based on other levels of evidence including open cohort studies and experienced clinical opinion. Patients at sufficiently low body weight may demonstrate dermatologic manifestations of starvation including alopecia, xerosis, hypertrichosis lanuginose, cheliosis, carotenoderma, pruritis, and nail fragility [1,2]. These changes are most apparent when the body mass index (BMI) drops below 16 [2].

Patients who induce vomiting will often do so mechanically by inserting their fingers into their mouths. Over time, introduction of the hand into the mouth results in repetitive trauma and skin abrasions to the hand, ultimately resulting in callous formation on the dorsal aspect of the hand. This characteristic finding is referred to as Russell’s sign [3].

### Eyes, ears, and nose

Self-induced vomiting may result in subconjunctival haemorrhage or recurrent epistaxis [4]. Subconjunctival haemorrhage consists of red patches in the white of the eyes, and although worrisome in appearance, is actually a benign finding. Recurrent bouts of epistaxis should prompt inquiry about purging.

### Dental

A patient’s dentist may be the first one to see signs of self-induced vomiting. Several abnormalities in the oral cavity have been reported including dental erosion, reduced salivary flow rate, tooth hypersensitivity, dental caries, periodontal disease, and xerostomia (dry mouth) [5-10]. Dental erosions typically occur on the lingual surface of the maxillary teeth. Though mandibular teeth may also be affected, they are believed to be somewhat protected, from gastric acid exposure, by the tongue [5,11]. Erosions may be apparent as early as six months after onset of regular self-induced vomiting [6]. The rate and severity of erosions may ultimately be determined by duration of illness, types of food consumed, oral hygiene, frequency of vomiting, and baseline quality of the tooth structure [11]. Increased frequency of dental caries has been reported as a consequence of binging on high carbohydrate-content foods, increased consumption of carbonated beverages, poor oral hygiene, in addition to acid exposure [7-9]. Gingivitis (gum disease) and periodontal disease may result from repeated exposure to gastric acid. This causes chronic gum irritation and bleeding. Xerostomia is encountered in patients with self-induced vomiting; it is hypothesized to relate to reduced salivary flow rates [10].

Other abnormalities, besides those affecting dentition, occur in the oral cavity as a consequence of self-induced vomiting. Sialadenosis, or hypertrophy of the salivary glands, has been reported in 10-50 percent of patients with self-induced vomiting [11]. It is generally bilateral and only minimally tender. Though the pathogenesis of this phenomenon is unclear, pathologic examination reveals a non-inflammatory process. Reductions in salivary flow have been observed but the electrolyte and protein composition of saliva does not differ between patients and controls [10]. It has been hypothesized that sialadenosis may be the result of either regurgitation of acidic contents, consumption of carbohydrate dense foods over a short period of time (binges), or the result of pancreatic proteolytic enzymes coming back into the mouth during vomiting and stimulating lingual receptors [12]. The bilateral parotid glands are the glands most commonly involved, but submandibular enlargement may also be seen [13,14]. This “chipmunk-type” facies generally occurs 3-4 days after the cessation of self-induced vomiting. The enlargement of salivary glands has been correlated with elevations in serum amylase levels. Kinzle, et al, found that 61 percent of bulimic patients, purging via self-induced vomiting, had elevated serum amylase levels [15]. Isoenzyme studies further demonstrate that the elevations in serum amylase originate from salivary glands as opposed to the pancreas [15-17]. Levels generally rise within 1-2 days of said purge episode and normalize within one week [11,17].

### Throat

Acid reflux, as a result of frequent bouts of self-induced vomiting and damage to the esophageal sphincters, affect areas of the pharynx and larynx and is referred to as laryngopharangeal reflux (LPR). Regurgitated acidic contents may come into contact with the vocal chords and surrounding areas, resulting in hoarseness, dysphagia, chronic cough, a burning sensation in the throat or repeated sore throats [18,19]. A study of eight singers with bulimia found that their throat examinations demonstrated some or all of the following: post cricoid edema, vocal fold edema, thick mucus covering the larynx, posterior commissure hypertrophy, ventricular obliteration, telangiectasia and polypoid changes in 50-100 percent of such patients [19].

### Gastrointestinal

Patients who induce vomiting will commonly complain of symptoms consistent with gastroesophageal reflux (GERD), dysphagia, and odynophagia [20,21]. These complaints generally imply abnormalities of the esophagus. With repetitive vomiting, the esophageal epithelium suffers repeated abnormal exposure to acidic gastric contents and microtrauma. Consequences of this can include esophagitis, esophageal erosions and ulcers, Barrett’s esophagus and bleeding. Barrett’s esophagus refers to a condition in which there is a change in the mucosal lining type due to chronic and repetitive abnormal acid exposure to the esophagus. It is a known risk factor for esophageal carcinoma. While the criteria to screen, via endoscopy, for Barrett’s esophagus have been recently loosened, screening may perhaps be indicated for bulimic patients with chronic esophageal reflux symptoms whose symptoms are difficult to control with acid reduction therapies [22]. The most severe, albeit very rare, acute consequence of self-induced vomiting, is Boerhaave’s syndrome (esophageal rupture); it is a surgical emergency [11,20]. This syndrome manifests with chest pain, shortness of breath, and the very unique complaint of painful yawning in a patient who is tachypneic, tachycardic and appears to be in significant distress. Overall, despite the potential frequency of complaints in these patients, endoscopic evaluation is generally normal or only demonstrates mild esophagitis [23]. Upper endoscopy may be indicated for those patients with bulimia who have purged excessively for years and for any bulimic patients with clear new symptoms of dysphagia. Esophageal motility studies typically have not demonstrated significant differences between patients and controls [20]. Why the frequency of gastrointestinal complaints does not correlate with objective endoscopic findings is not currently known.

### Electrolytes

Repeated episodes of vomiting can lead to dehydration and subsequent upregulation of the secretion of the renin-angiotensin-aldosterone steroid hormone system. Aldosterone is secreted by the adrenal glands and results in increased renal absorption of sodium and bicarbonate and subsequent water retention to mitigate against a propensity towards dehydration, hypotension and volume depletion from recurrent vomiting. This results in a metabolic alkalosis and low serum potassium values [24]. Taken together, this phenomenon is referred to as pseudo-Bartter’s syndrome [25]. Aldosterone continues to be upregulated even after purging ceases. The resultant ongoing avidity toward increased sodium and bicarbonate retention by the kidneys, in the absence of continued purging, can result in severe peripheral edema formation especially if the patient with bulimia is given intravenous saline-containing fluids in a rapid manner to correct dehydration or electrolyte abnormalities [4,26]. Additional potassium losses emanate from the actual vomitus. Though low serum potassium may be specific marker for the self-induced vomiting of bulimia, it is not sensitive [27,28]. The majority of patients with bulimia, who vomit only occasionally, will have normal serum electrolytes, in contrast to those who vomit excessively or those who do so very regularly for a protracted course of time.

### Cardiac

Dehydration as a result of repeated episodes of emesis can result in both resting and exertional sinus tachycardia, hypotension, and orthostasis. The resultant hypokalaemia can result in a prolonged QTc interval putting the patient at risk for significant arrhythmias resulting in syncope and palpitations. The most severe of these is a specific type of ventricular tachycardia known as torsades de pointes that can be fatal [29].

Though patients will often use their fingers or an object to induce emesis, some may revert to use of ipecac, a syrup previously used to treat acute toxic ingestions. Patients with bulimia who engage in self-induced vomiting may abuse this medication. The active ingredient of ipecac is emetine which has a long half-life and consequently can accumulate to toxic levels with chronic ingestion. Emetine toxicity can result in irreversible damage to cardiac myocytes resulting in severe congestive heart failure, ventricular arrhythmias, and sudden cardiac death [11,29].

### Reproduction

While not a direct result of self-induced vomiting, it is worth briefly mentioning that reproductive health outcomes are compromised in patients with bulimia. Although normal weight bulimics do not incur the difficulties with fertility experienced by patients with anorexia nervosa, bulimia nervosa has been associated in smaller cohort series with an increased risk of miscarriages [30].

### Pulmonary

In patients who purge via self-induced vomiting, aspiration of regurgitated food is a possibility. Thus, in an otherwise healthy young adult with sudden onset respiratory distress and lower lobe opacities on chest radiography, self-induced vomiting with aspiration should be considered. Another pulmonary complication of self-induced vomiting is pneumomediastinum, which is the dissection of air through the alveolar walls, due to retching [31]. Finally, the presence of an unusual foreign body in the esophagus or stomach on a chest radiograph, may be due to the accidental ingestion of an object like a toothbrush used to induce vomiting [32].

### Laxative abuse

While less common than self-induced vomiting, abuse of laxatives is the second most commonly utilized mode of purging in patients with bulimia nervosa. Laxatives can be grouped into five major classes, depending on their mechanism of action: bulk laxatives, osmotics, surfactants, emollients, and stimulants. Of the various classes of laxatives, the ones most abused by bulimic patients, and the ones associated with most of the medical complications, are the stimulant laxatives, including compounds containing phenolphthalein, senna, bisacodyl or anthraquinone. They act rapidly and directly to stimulate colonic motility, producing a large volume of watery diarrhea.

The medical complications of laxative abuse can be divided into two main categories, those due to effects on the gastrointestinal system along with the systemic effects, of hypovolemia and those due to electrolyte disturbances. The gastrointestinal effects of laxative abuse include melanosis coli, cathartic colon, and functional impairment. Melanosis coli is a dark brown discoloration of the colonic mucosa. Microscopic melanosis can be seen in about half of patients taking anthraquinone-based laxatives. There is no indication that melanosis coli has any significant pathophysiologic consequences.

In contrast, the cathartic colon syndrome is a serious entity, involving loss of normal colonic peristalsis because of long-term habituation to stimulant laxatives. The result is a dilated, atonic colon, which is incapable of propagating fecal material, typically defined on the basis of radiologic findings. A barium enema reveals that the colon loses the normal haustral markings and is dilated. Cathartic colon is suggested, on an abdominal radiograph, when an ahaustral colon is present with increased submucosal fat [33]. These changes arise from inflammation of the mucosa, alterations in muscular layers of the colon, and degeneration of the myenteric and Auerbach’s nerve plexi caused by a direct toxic effect from the stimulant laxatives. Microscopically, the colon shows thinning of the microvilli and abnormalities within cytoplasmic organelles. As a result of these changes, slowed or absent transit occurs through some or all segments of the colon, leading to hard, infrequently passed stools and refractory constipation wherein the colon is converted to an inert tube. There is marked variation in individual susceptibility to these effects of stimulant laxatives and a true prevalence of this devastating disorder in bulimia, is not known. With truly prolonged abuse of these laxatives, the cathartic colon syndrome is potentially irreversible. Loss of normal colonic function can become so severe that resultant ostomy is needed to treat intractable constipation with resultant ostomy. A history of prolonged laxative use may also produce a more innocuous reflex constipation. This constipation can be bothersome during withdrawal from laxatives, making it somewhat difficult to terminate laxative abuse, but it is usually transient if patience prevails.

The systemic effects of laxative abuse emanate from the hypovolemia and electrolyte disturbances that develop as a result of the diarrhea and the body’s compensatory mechanisms. Electrolytes lost through laxative use include chloride, calcium, bicarbonate, and potassium. The hypokalemia leads to further slowing of intestinal motility [34,35]. Moreover, the risk for severe edema formation with abrupt cessation of purging via laxatives can again occur with abrupt cessation of the laxatives [24]. Chronic diarrhea, like self-induced vomiting produces a hypochloremic, hypokalemic metabolic alkalosis attributable to hypovolemia-induced hyperaldosteronism, but acute diarrhea of shorter duration results in a hyperchloremic metabolic acidosis, without an increased anion gab (Table 1).

**Table 1:** **Common electrolyte changes with different purging modes**

***Purging mode***	***Sodium***	***Potassium***	***Chloride***	***Bicarbonate***
Diuretics	Decreased or normal	Decreased	Decreased	Increased
Laxatives (short-term)	Decreased or normal	Decreased	Increased	Decreased
Laxatives (long-term)	Decreased or normal	Decreased	Decreased	Increased
Vomiting	Decreased or normal	Decreased	Decreased	Increased

Because of the relatively high prevalence of laxative abuse in patients with bulimia, such abuse should be suspected and appropriate questions directed to these patients because bulimic persons are often of normal body weight and may not admit to their disorder or their laxative abuse. In suspected cases, laxative abuse can also be detected by ordering toxicological assays of the feces or urine, a way to establish a suspected diagnosis of laxative abuse beyond doubt. In addition, surreptitious laxative abuse should be suspected in those patients complaining of chronic diarrhea without an obvious source. In one study of patients being evaluated for diarrhea, the prevalence of laxative use was 15%, despite patients’ denying any laxative ingestion [36]. Restoration of normal bowel function may take weeks.

## Conclusions

Similar to anorexia nervosa, bulimia nervosa is associated with many different medical complications. These are dependent on the mode and frequency of purging. Diuretic abuse very closely mimics the acid-base and electrolyte abnormities seen with self-induced vomiting. Overall, some of these complications can be quite serious and can cause permanent adverse sequelae.

## Abbreviations

BMI: Body mass index
GERD: Gastroesophageal reflux
LPR: Laryngopharangeal reflux
